# Water Fraction Ratio of the Sacroiliac Joint Subchondral Bone Marrow in Patients with Ankylosing Spondylitis Predicts the Degree of Disease Activity

**DOI:** 10.3390/diagnostics12112842

**Published:** 2022-11-17

**Authors:** Beum Jin Kim, Young Han Lee, Joohee Lee, Sungjun Kim, Ho-Taek Song

**Affiliations:** Department of Radiology, Research Institute of Radiological Science, Center for Clinical Imaging Data Science (CCIDS), Yonsei University College of Medicine, Seoul 03722, Republic of Korea

**Keywords:** ankylosing spondylitis, ankylosing spondyloarthritis, sacroiliac joint, sacroiliitis, magnetic resonance imaging, mDixon, fat water separation, fat fraction, disease activity

## Abstract

Objectives: Ankylosing spondylitis (AS) is a chronic inflammatory arthritis with characteristic involvement of the spine and sacroiliac joints. MRI may be the only indicator of disease activity or response. This study aimed to use a novel water fraction measurement technique on MRI as a biomarker to predict disease activity in patients with AS. Methods: We enrolled 39 patients (18 men [mean age, 38.6 years; range, 18–59 years] and 21 women [mean age, 39.3 years; range, 23–61 years]) who were clinically diagnosed with AS and underwent MRI, including mDixon sequences. Water fraction values of sacroiliac joint subchondral bone marrow were derived from the mDixon sequences. The Ankylosing Spondylitis Disease Activity Score (ASDAS) was recorded using clinical information and laboratory values from medical records. Multiple linear regression, Firth logistic regression, and intraclass correlation coefficients were used for the statistical analysis. Results: In multiple linear regression, water fraction, subchondral bone marrow edema, subchondral bone erosion, and subchondral bone marrow enhancements were significantly associated with ASDAS with C-reactive protein (ASDAS-CRP). The water fraction parameters showed a good linear correlation with ASDAS-CRP and ASDAS with erythrocyte sedimentation rate (ASDAS-ESR) (beta coefficient = 1.98, *p* < 0.001 and beta coefficient = 1.60, *p* = 0.003). Firth logistic regression showed that water fraction was a significant predictor of ASDAS-CRP but not ASDAS-ESR. The intraclass correlation coefficient showed excellent repeatability for the three repeated measures of the water fraction. Conclusion: Water fraction parameter could be a good imaging biomarker of disease activity status. The sacroiliac joint evaluated by mDixon MRI may be a promising biomarker of disease progression in patients with spondyloarthritis.

## 1. Introduction

Ankylosing spondylitis (AS) is a chronic inflammatory arthritis characterised by involvement of the spine and sacroiliac joints [1]. The disease burden is rather high due to its young age at onset and delayed diagnosis [2]. In these patients, early detection and initiation of treatment are key to improving long-term functional outcomes [3]. AS is diagnosed using a combination of clinical, serological, and imaging features [4]. Clinical criteria include the presence of inflammatory-type low back pain and other features of AS, such as anterior uveitis, serological criteria related to human leukocyte antigen (HLA) B27 positivity, and imaging criteria related to imaging evidence of sacroiliitis and spondyloarthritis [5].

The disease persistently alternates between active and chronic stages; thus, identifying the level of disease activity is fundamental in clinical practice to monitoring disease activity and setting thresholds for biologic treatment [6]. While conventional laboratory inflammatory markers such as elevated C-reactive protein (CRP) and erythrocyte sedimentation rate (ESR) can provide objective information, their sensitivity and specificity are unsatisfactory [6]. The Ankylosing Spondylitis Disease Activity (ASDAS), the most recently developed index to assess disease activity in AS, is recommended for treatment response follow-up [7]. A higher score indicates higher activity, and a score less than 1.3 is considered an inactive disease state, the ultimate treatment target. Two versions of the ASDAS have been defined: the ASDAS-CRP (with CRP) and the ASDAS-ESR (with ESR); the index is commonly used to assess patients with AS since it provides disease activity level [8].

Imaging is a key in the diagnosis and management of AS. Radiography is the conventional gold standard method for assessing structural changes, whereas magnetic resonance imaging (MRI) detects early changes in sacroiliitis that may not be visible on plain radiographs [9]. Therefore, early detection using MRI is vital. Scoring systems for MRI of the sacroiliac joints have been developed to enable a more systematic and reliable assessment of active inflammation in patients with AS; however, it is subjective and prone to inter-reader bias. The definition of a positive MRI finding is visible bone marrow edema in subchondral bone on T2-weighted images or the presence of osteitis [10]. Positive MRI findings are considered treatment targets and treatment monitoring markers. MRI can also provide other structural features of sacroiliitis, such as bone erosion, ankylosis, and fatty metaplasia [11].

Unfortunately, the quantitative evaluation of active inflammation on MRI remains limited. A method that can objectively and reliably evaluate bone marrow inflammation to support diagnostic and therapeutic decisions is necessary. Previous studies investigated the use of chemical shift–encoded MRI in other parts of the body as objective methods to quantitatively assess fatty deposits and bone marrow edema in subchondral bone [12]. The water fraction (WF) is a simple and direct visualisation method of water signals from the fat-water separation technique mDixon MRI, allowing direct communication of the amount of water within the subchondral bone marrow edema [13]. This may further indicate disease activity severity in AS. The purpose of this study was to analyse the correlation between the WF ratio of the sacroiliac joint subchondral bone marrow on water-fat separation MRI sequences and evaluate imaging features to predict disease activity in patients with AS.

## 2. Methods

### 2.1. Study Population

This retrospective study received approval from our institutional review board, which waived the requirement for informed consent. From January 2016 to December 2020, 285 patients who were clinically diagnosed with AS and underwent sacroiliac joint MRI were retrospectively recruited. The inclusion criteria were previous diagnosis of AS according to the Assessment of Spondyloarthritis International Society (ASAS) classification criteria for axial spondylitis and having undergone sacroiliac joint MRI containing full mDixon sequences (n = 53). Fourteen patients were excluded: five with inadequate laboratory test results and nine with inadequate medical records, preventing the ASDAS calculation. Clinical characteristics including age, sex, back pain severity, morning stiffness duration, global symptom duration, history of uveitis, and peripheral arthritis were investigated. Human leukocyte antigen-B27 positivity, serum ESR, and CRP levels were obtained from the patients’ electronic medical records. ASDAS-ESR and ASDAS-CRP were calculated at the time of the imaging study.

### 2.2. Imaging Protocol and Reconstruction

MRI was performed using the same vendor scanner as the 3-T MRI scanner (Ingenia^®^, Ingenia Cx^®^, Achieva^®^, Achieva TX^®^; Phillips Healthcare, Best, The Netherlands). The axial and coronal images were processed using the mDixon water/fat separation algorithm using two echo variants and a bone marrow model. After the MRI acquisition, each image was automatically reconstructed as in-phase, opposed-phase, water images, and fat images. The following parameters were used for mDixon T2 images: repetition time = 3000–3700 ms; echo time = 80 ms; number of excitations = 1; echo train length = 6; field of view = 240–380 mm; matrix size = 256 × 256 pixels; section thickness, 2 mm; intersection gap, 2 mm; and flip angle, 50°. Multi-coils were used for all the images (built-in posterior spine array coil combined with anterior coil on Ingenia MRI scanners; torso coil combined with posterior spine receiver coils on Achieva MRI scanners).

### 2.3. Image Analyses

**Figure 1:**
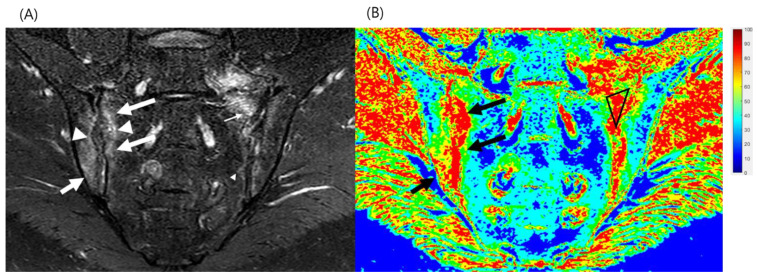
A 56-year-old man diagnosed with ankylosing spondylitis presented with a 2-month history of worsening back pain, morning stiffness, and right-sided Achilles enthesitis. His C-reactive protein level was 74.9 mg/L, while his erythrocyte sedimentation rate was 120 mm/hr. Human leukocyte antigen-B27 was positive. His Ankylosing Spondylitis Disease Activity Score with C-reactive protein was 3.70 and Ankylosing Spondylitis Disease Activity Score with erythrocyte sedimentation rate was 4.17, indicating a very high disease activity state. (**A**) An mDixon T2 coronal water image showing subchondral bone marrow edema (arrows) and erosions (arrow heads) in both sacroiliac joints. (**B**) Water fraction map of the sacroiliac joint on mDixon magnetic resonance imaging. The normal marrow regions are blue or green, and the active inflammatory regions (edema) are orange. A polygonal-shaped region of interest over the subchondral bone marrow edema was established to evaluate the water fraction. The mean water fraction value was 0.866.

**Figure 2:**
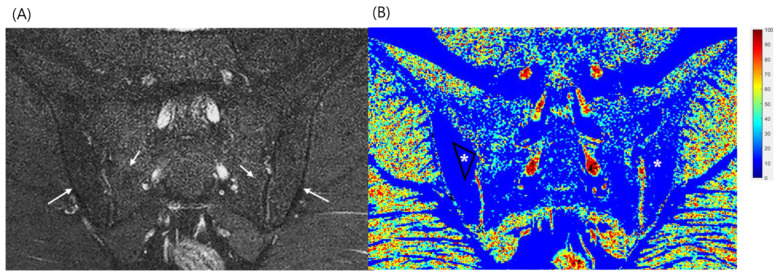
A 25-year-old man previously diagnosed with ankylosing spondylitis presented for regular follow-up after medical therapy. The patient’s symptoms of pain and stiffness had improved. His Ankylosing Spondylitis Disease Activity Score with C-reactive protein was 1.04 and his Ankylosing Spondylitis Disease Activity Score with erythrocyte sedimentation rate was 0.93, indicating an inactive disease state. (**A**) An mDixon T2 coronal water image showing no definite subchondral bone marrow edema. Fatty changes in both sacroiliac joints are noted (arrows). (**B**) A water fraction map of the sacroiliac joint on mDixon images. The fatty regions are blue (asterisk). A polygonal-shaped region of interest over the fatty bone marrow was established to evaluate the water fraction. The mean water fraction (n = 3) value was 0.158.

Sacroiliac joint subchondral bone marrow fat signal and water signal fractions were quantified using mDixon sequences. The WF and fat fraction (FF) were measured using the WF map, and the measurements represented the percentage of fat and water content in the bone marrow. The WF was calculated by dividing the signal intensity of the water-only image by the sum of the fat-only and water-only image signal intensities: S_water_/(S_fat_ + S_water_) [12,13]. Coronal plane slice showing largest bone marrow edema was selected and WF parameters were independently measured using manually drawn polygonal regions of interest (ROIs) that were as large as possible within the selected target bone marrow edema on the WF map by a single radiologist. Relevant MRI features such as joint erosion and ankylosis, subchondral bone marrow sclerosis, fatty infiltration and enhancement were recorded. Same process was repeated three different separate times with randomly mixed order of patients and mean value was used for analysis. A normal bone marrow signal in the ilium and sacrum, which are far from the SI joint, was considered normal marrow fat. When a subchondral region around sacroiliac joint had high signal intensity on both T1- and T2-weighted images, it was considered a post-inflammatory fat deposition area. When an area had low signal intensity on a T1-weighted image and high signal intensity on a fat-suppressed T2-weighted image, it was considered bone marrow edema. Drawing of polygonal WF ROIs and calculation of WF were performed using in-house developed software written in MATLAB (Mathworks, Natick, MA, USA; Figure 1 and Figure 2). The WF was calculated by dividing the signal intensity of the water-only image by the sum of the fat-only and water-only image signal intensities: S_water_/(S_fat_ + S_water_).

### 2.4. Statistical Analyses

All data are expressed as mean ± SD and percentage. The reliability of ROI for WF was assessed by repeating the measurements three separate times. The intraclass correlation coefficient (ICC) was used to evaluate WF measurement repeatability. The association between WF and other multiple variables with ASDAS was assessed using multiple linear regression analyses. Firth logistic regression analysis was used to analyse the relationship between variables and active disease state (ASDAS > 1.3). All statistical analyses were performed using R software (version 4.0.5; R Foundation for Statistical Computing, Vienna, Austria). For all assessments, values of *p* < 0.05 were considered statistically significant.

## 3. Results

**Figure 3:**
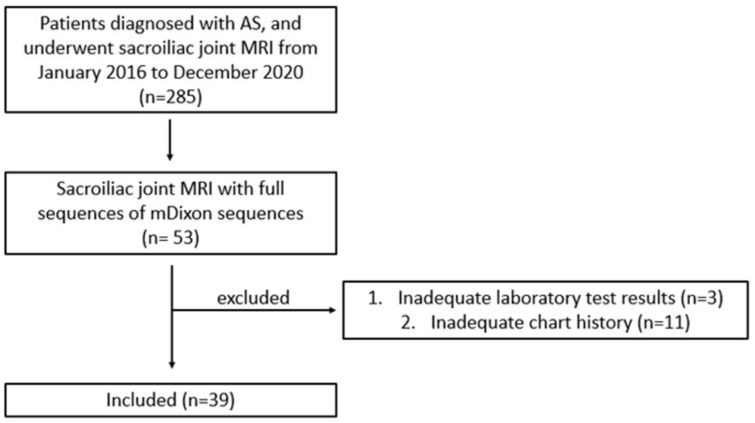
Flowchart showing the patient selection process for the study.

A total of 39 patients (18 men [mean age, 38.6 years; range, 18–59 years] and 21 women [mean age, 39.3 years; range, 23–61 years]) were included in the study (Figure 3).

Table 1 summarises the baseline patient characteristics. The mean patient age was 39.1 ± 14.4 years.

The mean ASDAS-CRP and ASDAS-ESR evaluated at the time of MRI were 1.80 ± 0.80 and 2.20 ± 0.84, respectively. AS patients with an ASDAS-CRP > 1.3 (defined as active state) had significantly higher mean WF values (0.484 ± 0.256) than those in the inactive group (0.268 ± 0.138). In the multiple linear regression, WF, bone marrow edema, bone erosion, and bone marrow enhancements in subchondral bone were significantly associated with ASDAS-CRP and ASDAS-ESR (Table 2).

**Figure 4:**
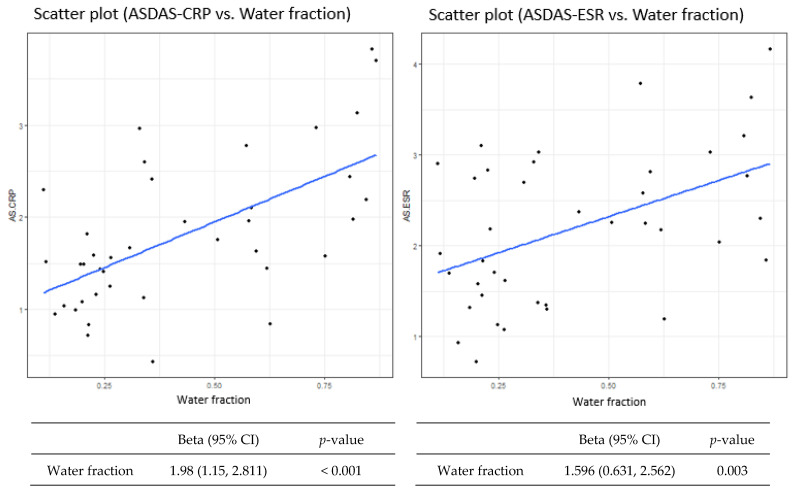
Scatter plot of Ankylosing Spondylitis Disease Activity Score with C-reactive protein or erythrocyte sedimentation rate (ASDAS-CRP/ASDAS-ESR) vs. water fraction.

The WF parameters also showed a good linear correlation with ASDAS-CRP and ASDAS-ESR (beta coefficient = 1.98 [95% confidence interval (CI), 1.15–2.81], *p* < 0.001; and beta coefficient = 1.60 [95% CI, 0.63–2.56], *p* = 0.003) (Figure 4).

Firth logistic regression showed that WF was a significant predictor of ASDAS-CRP but not ASDAS-ESR (Table 3).

Bone erosion was also significantly correlated with ASDAS-CRP and ASDAS-ESR. The intraclass correlation coefficient showed excellent repeatability of the three repeated measures of the WF (beta coefficient = 0.961 [95% CI, 0.933–0.978]).

## 4. Discussion

This study quantified sacroiliac joint subchondral bone marrow edema using the mDixon MRI method and investigated its clinical significance as an imaging biomarker of active inflammation. The WF ratio of sacroiliac joint subchondral bone marrow edema showed a strong positive correlation with the ASDAS-CRP and ASDAS-ESR. Bone erosions, bone marrow edema, and enhancements in subchondral bone also showed a positive correlation with ASDAS-CRP and ASDAS-ESR. This study’s findings suggest that the WF ratio derived from mDixon MRI is a significant predictor of an active disease state.

Previous studies demonstrated the usefulness of quantifying fat in the bone marrow by measuring the FF of patients with sacroiliitis in AS [14,15,16,17,18]. Similar to our study, they all showed low FF values in bone marrow edema and high FF values in normal or chronic states. Proton density FF measurements showed changes in marrow composition in areas of bone marrow edema and fat metaplasia and was previously used to monitor both active inflammation and structural damage in spondyloarthritis. In our study, T2 mDixon MRI was used, which is more efficient on sacroiliac joint imaging. Conventional imaging and quantitative WF imaging can be obtained simultaneously within one sequence: in-phase T2-weighted images and water images of fluid-sensitive sequences as well as WF. Quantitative MRI reportedly provides more accurate differentiation of bone marrow edema and fatty metaplasia.

Recent studies also investigated the usefulness of apparent diffusion coefficient (ADC) values from diffusion-weighted MRI, as they are influenced by bone marrow edema [19,20]. However, this technique has limitations including low resolution, geometric distortion, signal-intensity dropout, and T2*-induced blurring, which disable accurate localisation of active lesions on MRI [21]. A single study investigated the association between the FF of the sacroiliac joint and chronicity of the disease using radiographic staging and showed similar results to those of our study [18]. Another recent study showed similar results to our study, showing that the Dixon sequence water-fat ratio of the bilateral sacroiliac joints positively correlated with SPARCC (Spondyloarthritis Research Consortium of Canada), BASFI (Bath ankylosing spondylitis functional index) and BASDAI (Bath ankylosing spondylitis disease activity index) scores [22].

As observed in our study, subchondral bone erosion showed a strong positive correlation with ASDAS and a significant predictor of disease activity. Bone erosion at the sacroiliac joint surface seems a reasonable indicator for inclusion in the definition of sacroiliitis on MRI, which is also supported by our study. The dilemma is that international consensus is lacking on how erosion should be defined on MRI or quantified, which warrants additional research in the future [10].

The enhancement of subchondral bone marrow also showed significant correlations with ASDAS, suggesting the usefulness of contrast-enhanced imaging in sacroiliac joint evaluations, although UK guidelines suggest that short tau inversion recovery (STIR) images are sufficient for detecting inflammation [23]. The mDixon MRI method requires a shorter examination time and lower signal-to-noise ratio and is more effective at suppressing fat signals than conventional STIR images. Thus, in combination with WF values, the mDixon MRI method may be a more useful tool for assessing disease activity in patients with AS.

This study had several limitations. First, selection bias was inevitable because of its retrospective study design and because the patients were collected from a single territory centre. Second, ASDAS consists of both subjective questionnaires and objective laboratory inflammatory markers, and both can be affected by various other co-existing medical conditions. Third, the analysis did not include bone mineral density, an important factor affecting FF and WF. Moreover, most patients were in an active disease state, and an insufficient number of patients were inactive. However, MRI is usually performed in symptomatic patients, so this seems inevitable in such studies.

In conclusion, WF-related parameters could be a good imaging biomarker for predicting AS severity. We discovered that sacroiliac joint subchondral bone marrow edema WF was strongly positively correlated with ASDAS and can, thus, predict disease activity. Therefore, the complementary use of WF with other structural changes identified on MRI and perhaps in combination with ADC values may provide additional quantitative and objective information about AS disease activity. We propose the use of the mDixon method as a useful tool for assessing disease activity in patients with AS. WF could be utilised in combination with conventional disease activity evaluations for therapeutic planning and treatment monitoring in AS patients.

**Key points:**

- Water fraction parameters can be derived from mDixon MRI and can provide quantitative parameter of disease activity in ankylosing spondylitis patients.
- Retrospective study showed good correlation between the water fraction parameters with ASDAS scores.
- Water fraction could be utilized for therapeutic planning and treatment monitoring in ankylosing spondylitis patients when combined with conventional disease activity evaluations.

## Figures and Tables

**Table 1:** Baseline characteristics of the study population.

	Total (N = 39)
Age, mean ± SD years	39.1 ± 14.4
Male, n (%)	18 (46.2)
HLA-B27 positivity, n (%)	20 (51.3%)
CRP, mean ± SD	6.98 ± 14.3
ESR, mean ± SD	24.4 ± 28.2
ASDAS-CRP, mean ± SD	1.80 ± 0.80
ASDAS-ESR, mean ± SD	2.20 ± 0.84
Water fraction, mean ± SD	42.3 ± 24.8
Bone erosions, n (%)	15 (38.5%)
Fatty infiltrations, n (%)	10 (25.6%)
Sclerosis, n (%)	15 (38.5%)
Ankylosis, n (%)	3 (7.7%)
Joint space alterations, n (%)	8 (20.5%)
Bone marrow enhancement, n (%)	13 (33.3%)
Joint effusion, n (%)	1 (2.6%)
Enthesitis, n (%)	5 (12.8%)
Uveitis, n (%)	4 (10.3%)

**Table 2:** Multiple linear regression analysis: ASDAS-CRP and ASDAS-ESR.

	ASDAS-CRP		ASDAS-ESR	
Variable	Beta (95% CI)	*p* value	Beta (95% CI)	*p* value
Age	0.011 (−0.006 to 0.029)	0.218	0.019 (0.002–0.037)	0.039 *
Sex	0.229 (−0.279 to 0.737)	0.382	−0.27 (−0.799 to 0.259)	0.324
Bone marrow oedema	0.565 (0.085–1.045)	0.027 *	0.566 (0.062–1.07)	0.034 *
HLA B27	−0.001 (−0.513 to 0.511)	0.998	0.047 (−0.488–0.581)	0.865
Fatty metaplasia	−0.204 (−0.786 to 0.378)	0.497	−0.266 (−0.872 to 0.34)	0.395
Bone Erosion	0.773 (0.31–1.237)	0.002 *	0.791 (0.304–1.278)	0.003 *
Sclerosis	0.153 (−0.371 to 0.677)	0.57	0.092 (−0.456 to 0.641)	0.743
Ankylosis	0.659 (−0.278 to 1.595)	0.176	0.576 (−0.41 to 1.562)	0.26
Joint space alteration	0.086 (−0.547, 0.719)	0.791	0.071 (−0.591, 0.732)	0.836
Bone marrow enhancement	0.589 (0.081, 1.098)	0.029 *	0.631 (0.101, 1.16)	0.025 *
Joint space fluid	−0.981 (−2.569 to 0.607)	0.233	−1.034 (−2.692 to 0.624)	0.229
Enthesitis	0.48 (−0.27–1.229)	0.218	0.576 (−0.202 to 1.354)	0.155
Uveitis	0.198 (−0.643 to 1.039)	0.647	0.425 (−0.445 to 1.296)	0.344

* Significant correlation at the 0.05 level.

**Table 3:** Firth logistic regression analysis: ASDAS-CRP > 1.3 and ASDAS-ESR > 1.3.

	ASDAS-CRP > 1.3		ASDAS-ESR > 1.3	
Variable	Odds ratio (95% CI)	*p* value	Odds ratio (95% CI)	*p* value
Age	1.024 (0.972–1.079)	0.374	1.025 (0.959–1.095)	0.468
Sex	1.04 (0.256–4.218)	0.956	0.368 (0.059, 2.302)	0.285
Bone marrow oedema	3.077 (0.673–14.077)	0.147	1.882 (0.302–11.728)	0.498
Water fraction	177.68	0.028 *	15.222	0.239
HLA B27	0.495 (0.118–2.081)	0.337	1.062 (0.187–6.052)	0.946
Fatty metaplasia	0.889 (0.183–4.31)	0.884	0.269 (0.044–1.637)	0.154
Bone Erosion	26.407 (1.292–539.639)	0.001 *	10.892(0.517–9.567)	0.036 *
Sclerosis	0.667 (0.162–2.748)	0.575	1.3 (0.207–8.148)	0.779
Ankylosis	3.157 (0.096–103.877)	0.404	1.492 (0.044–50.786)	0.791
Joint space alteration	1.227 (0.207–7.265)	0.821	0.444 (0.066–3.014)	0.406
Marrow enhancement	2.912 (0.527–16.093)	0.22	1 (0.158–6.329)	>0.999
Joint space fluid	0.123 (0.001–12.008)	0.169	0.055 (0.001–5.541)	0.062
Enthesitis	5.383 (0.208–138.985)	0.179	0.69 (0.063–7.512)	0.76
Uveitis	1.2 (0.111–12.953)	0.881	0.5 (0.043–5.813)	0.58

ASDAS, Ankylosing Spondylitis Disease Activity Score; CRP, elevated C-reactive protein; ESR, erythrocyte sedimentation rate; HLA, human leukocyte antigen Asterisks (*) indicate statistical significance (*p* < 0.05).

## Data Availability

The datasets generated during and/or analyzed during the current study are available from the corresponding author on reasonable request.

## Abbreviations

AS: Ankylosing spondylitis; ASDAS: Ankylosing Spondylitis Disease Activity Score; ASAS: Assessment of Spondyloarthritis International Society; WF: Water fraction; FF: Fat fraction; CRP: C-reactive protein; ESR: Erythrocyte sedimentation rate.
